# Factors associated with burnout in Polish healthcare workers during the COVID-19 pandemic

**DOI:** 10.3389/fpubh.2022.1018612

**Published:** 2023-01-04

**Authors:** Paweł Holas, Natalia Wojtkowiak, Małgorzata Gambin, Karolina Hansen, Grażyna Kmita, Ewa Pisula, Kamilla Bargiel-Matusiewicz, Emilia Łojek

**Affiliations:** Faculty of Psychology, University of Warsaw, Warsaw, Poland

**Keywords:** COVID-19, pandemic, burnout, empathy, healthcare workers, mental health, anxiety, depression

## Abstract

**Introduction:**

The COVID-19 pandemic has placed the healthcare system under substantial strain that has caused elevated psychological distress among healthcare workers (HCWs). Previous studies have found a high prevalence of burnout among HCWs exacerbated by the COVID-19 pandemic and have delineated some associated factors, but further research is needed. Little is known, for example, whether the economic status of HCWs or experiencing negative and positive emotions contribute to burnout. The present study was meant to fill this gap.

**Methods:**

A total of 412 HCWs (i.e.: nurses - 47%, physicians-28%, psychologists-14%, and other healthcare professionals-11%), aged 21–69 years (*M* = 36.63; SD = 11.76) participated in a web-based cross-sectional study. Data was collected from June to November 2020. The participants filled out measures assessing two dimensions of burnout (Exhaustion and Disengagement), depression, generalized anxiety, positive and negative emotions, along with the survey evaluating organizational aspects of their work during the pandemic.

**Results:**

Burnout thresholds were met by 54 and 66% of respondents for Disengagement and Exhaustion, respectively, which is high but comparable to levels found in other countries during the pandemic. Similarly to previous work, depression and anxiety were high in HCWs, with 24 % of them being in the risk group for clinical severity of depression and 34% in the risk group for a clinical generalized anxiety disorder (GAD). Regression analysis showed that the intensity of negative emotions was the strongest predictor of Exhaustion, whereas the intensity of positive emotions was the strongest predictor of Disengagement. Depression and GAD symptoms were positively related to Exhaustion, and economic status was inversely related to Disengagement.

**Discussion:**

These results suggest that distress in HCWs during the pandemic was related to symptoms of burnout, whereas higher income and experiencing positive emotions were associated with reduced burnout levels. Our findings call for the development of burnout intervention programs that could build capacities for dealing with depression and other negative emotions and at the same time teach skills on how to increase positive emotions in HCWs.

## Introduction

Burnout is described as a self-reported state of care- or work- related physical and mental stress (1), which consists of three-dimensional affective response: emotional Exhaustion, depersonalization, and a loss of sense of personal accomplishment (2, 3). According to other conceptualizations, burnout is composed of two elements: “Exhaustion”, which is related to excessive work demand; and “Disengagement” linked to insufficient job resources (4). Burnout has a negative impact on healthcare staff as well as patients (5, 6) and is of crucial importance to effective healthcare delivery, in particular at the challenging times.

The coronavirus disease 2019 (COVID-19) pandemic has led to a significant global public health crisis with an unprecedented strain on the healthcare system. The COVID-19 pandemic had a serious impact on health systems imposing significant changes in healthcare delivery, including cessation of routine services, repurposing of clinical areas and redeployment of staff (7). During the pandemic, healthcare workers (HCWs) experienced various kinds of stressors, including: unprecedented demands, extensive responsibilities, shortage of personal protective equipment, and constant risk of complaints for negligence (8, 9). Moreover, healthcare workers struggled with deterioration of some of the job resources e.g., COVID-19 restrictions led to reduced social connectedness at work due to closure of community spaces, cancellation of gatherings designed for celebration and connection with others or improving knowledge and professional skills (10). It is no surprise, therefore, that studies showed an increased level of burnout in HCWs during COVID-19 pandemic (11–13), as well as elevated risk of mental health problems such as depressive and anxiety disorders, PTSD, suicidal ideation (14–16) and also cognitive impairments due to COVID-19 (17). Research showed that previous infectious diseases outbreaks, such as severe acute respiratory syndrome (SARS) and Middle East Respiratory Syndrome (MERS), also evidenced their negative effects on the psychological well-being of HCWs, including burnout (18). In comparison to previous pandemic, however, the psychological impact of the COVID-19 pandemic is even more pronounced and widespread (19).

In the current research we focus on the factors associated with burnout in Polish healthcare workers. On March 15th 2020 a national lockdown started in Poland following the first case of COVID-19 reported in the country on March 4th 2020. Twenty three hospitals were turned into infectious diseases hospitals, and 67 of hospitals had infectious disease wards dedicated to cases of COVID-19. In spite of the fact that HCWs in Poland and other countries were subjected to significant burdens, there is paucity of studies assessing burnout level and its predictors. In the advent of the COVID-19 pandemic, research into burnout among HCWS has emerged, still, further investigation into the determinants of burnout in HCWs during pandemic is warranted. The only pre-pandemic study in Poland using OLBI as a tool for burnout in professions of social services (*N* = 1804) including HCWs (*N* = 491) showed mean Exhaustion score of 2.31 and the mean score of 2.38 for Disengagement (20). Several factors were proposed to have contributed to burnout of HCWs during COVID-19 pandemic, including persistent stress, excessive workload, concerns about infections and health of the families, as long as clinical role of HCWs, being redeployed, and levels of anxiety and depression (8, 13). Previous studies revealed that burnout, anxiety and depression are distinct constructs that are reciprocally related to each other (21, 22). Much less is known about the relation of economic status and experienced emotions to burnout.

The aims of the current study were twofold. First, we aimed at assessing the level of burnout and its dimensions in HCWs during COVID-19 pandemic in Poland in comparison to burnout levels reported in other countries. In addition, we wanted to assess if different HCWs groups differed in terms of burnout and mental health indicators and establish how many of them fulfilled the criteria for clinical risk of depression and generalized anxiety. Second, we aimed at evaluation of economic status, and emotions, in addition to depressive and anxiety symptoms as predictors of the two dimensions of burnout. We expected that there would be a high level of burnout among all HCWs groups, similarly as in other countries during the pandemic. Furthermore, we hypothesized that intensity of negative emotions along with depression and anxiety would positively predict both dimensions of burnout, whereas positive emotions and economic status would be negatively related to them.

## Methods

### Participants

The study link was clicked by 461 participants, of whom 412 indicated their profession. Out of the 412, 306 participants completed all questionnaires. The participants were aged 21–69 years (*M* = 36.63, *SD* = 11.76) and were recruited to the study by advertisements in medical portals and dedicated mailing to health professionals. The majority of the sample were women (88%), workers with higher education level (95%), and people living in large (i.e., over 500 000 habitants, 33%) or medium size cities (i.e., 100 000 habitants, 28%). In terms of profession, nurses constituted the largest group of surveyed specialists (47%), followed by physicians (28%), psychologists (14%), and other healthcare professionals (11%). When asked about the workplace, the participants mentioned various types of institutions and 32% indicated more than one type of workplace. Substantial number of the HCWs (21%) worked in facilities dedicated to COVID-19 patients as a result of transformation of the current workplace or as a consequence of being delegated to a different healthcare facility. About one third of the surveyed professionals (32%) worked remotely and almost one fifth (19%) experienced COVID-19 infection or it was highly probable that they had been infected before or at the time of conducting the study. Detailed information about the participants is presented in Table 1.

**Table 1:** Detailed information about participants of the study.

	**Overall HCWs**		**Physicians**	**Nurses**	**Psychologists**	**Other HCWs**
	***N***	**%**	**%**	**%**	**%**	**%**
**Gender**	
Female	378	89	75	97	93	84
Male	48	11	25	3	7	14
Other/refused to answer	1	0	0	0	0	2
**Education**	
Secondary	6	1	0	2	0	7
Post-secondary	17	4	4	3	0	14
BA	127	30	2	61	0	2
MA or PhD	274	65	94	34	100	77
**Economic status**	
Very bad	3	0	2	1	0	0
Rather bad	11	3	3	4	0	0
Hard to define	46	11	8	10	13	9
Rather good	135	32	21	38	30	39
Good	180	42	48	40	43	36
Very good	51	12	18	8	14	16
**Place of residence**	
Village	60	14	16	15	9	11
City < 20K of habitants	28	6	4	8	5	11
City < 99K of habitants	75	18	13	21	11	14
City < 500K of habitants	121	29	17	34	37	27
City >500K of habitants	142	33	50	22	38	37
**Workplace (multiple choice)**	
Public facility	112	27	38	21	23	34
Non-public facility	80	19	33	10	27	16
Health center	81	19	33	9	32	11
Hospital ward	218	52	47	64	46	25
Isolation ward	8	2	3	2	2	0
Emergency ward	9	2	4	1	0	2
Private medical practice	59	14	35	3	14	9
Other facility	46	11	5	10	7	30
**Type of facility**	
Dedicated or transformed into facility dedicated to COVID-19	87	21	21	21	16	28
**Type of work**	
Remote	129	32	60	12	48	26
On-site	258	63	38	81	52	58
Not applicable	21	5	2	7	0	16
**COVID-19 infection status**	
Infected/probably infected during the research	33	8	6	12	2	2
Infected/probably infected in the past	47	12	11	15	11	2
Never infected	234	57	57	54	67	61
Unknown	96	23	26	19	20	35

### Procedure and measures

The current study was a part of a wider research concerning experiences and psychological aspects of the pandemic among healthcare workers. All data were collected from July to November 2020, which coincided with the beginning of the second wave of the pandemic in Poland and the period immediately preceding its start. The research was approved by the institutional review board of the Faculty of Psychology, University of Warsaw. The survey was conducted using an online questionnaire, which was shared in Polish medical portals and social media groups dedicated to healthcare professionals.

The opening part of the survey included questions related to demography and participants' individual and professional situation such as economic status. Economic status was assessed subjectively with the following question: “Overall, how do you assess your family's current financial situation?” on a six-point scale from very bad to very good (see Table 1 for detailed information about other demographic measures). Following parts consisted of, among others, measures of burnout [Oldenburg Burnout Inventory, (23)], general anxiety [The Generalized Anxiety Disorder-7 questionnaire, (24)], depression [The Patient Health Questionnaire-9, (25, 26)], and positive and negative emotions [Questionnaire of Emotional State based on Lazarus and Folkman's theory, (27, 28)].

#### The Oldenburg burnout inventory

The Oldenburg Burnout Inventory (OLBI) is a 16-item questionnaire to assess burnout (23) with two dimensions: Exhaustion and Disengagement. Exhaustion describes feelings of emptiness, physical Exhaustion, overwork, and a strong need for rest, whereas Disengagement refers to distancing oneself from the objects and content of one's work. Each dimension consists of eight items rated on a 4-point Likert scale with both positively and negatively worded questions. Cronbach's alpha was α = 0.72 for Disengagement and, α = 0.76 for Exhaustion in the current study.

#### The patient health questionnaire-9

The Patient Health Questionnaire-9 [PHQ-9; (25, 26); Polish version: www.phqscreeners.com] is a screening tool for assessing the risk of depressive disorders. It consists of nine basic items that refer to the frequency of depressive symptoms (described in the DSM-IV and DSM-V diagnostic criteria) in the last 2 weeks. The participants give answers on a scale from 0—not at all to 3—nearly every day (in our study α = 0.91).

#### The generalized anxiety disorder-7

The Generalized Anxiety Disorder-7 questionnaire [GAD-7; (24); Polish version: www.phqscreeners.com] is a screening measure for assessing risk of generalized anxiety disorder. It consists of seven items about the frequency of symptoms during the last 2 weeks. The participants answer on a scale from 0—not at all to 3—nearly every day (α = 0.94).

#### Questionnaire of emotional state

Questionnaire of Emotional State [QES, (28, 29)] is a 15-item measure constructed by Heszen-Niejodek et al. (28) on the basis of a similar tool, initially introduced by Folkman and Lazarus (27). It contains a list of 15 adjectives describing different emotional states.

Exploratory factor analysis was performed. The eigenvalues acquired for consecutive solutions showed that only the first two values fulfilled the Kaiser criterion, i.e., were higher than the cut-off value equal to 1. The first eigenvalue was equal to 6.81, the second one was equal to 2.69 and the third one was equal to.81. Therefore, two factors were extracted. Next, the reliability of the extracted factors was verified. Cronbach's alpha was α = 0.91 for Negative Emotions (e.g., anger, disappointment, helplessness), and α = 0.91 for Positive Emotions (e.g., hope, optimism, enthusiasm).

### Statistical analysis

Firstly, the level of Disengagement and Exhaustion and their clinical thresholds were computed. Next, the groups of nurses, physicians, psychologists, and other health professions were compared with the use of one-way ANOVA. The analyzed variables did not differ significantly from normal distribution in terms of skewness and kurtosis. However, the groups compared were not equal. Therefore, one-way analysis of variance was followed by Gabriel *post-hoc* test. With the use of linear regression analysis we analyzed the relationships between professional burnout and positive and negative emotions, depressive symptoms, GAD symptoms, economic status, and variables describing reorganization at work (such as redeployment to a different facility). Conventional cut-off point of.05 for statistical significance was applied. In all statistical analyses the cases with missing values were excluded pairwise.

## Results

### The level of burnout

The average OLBI scores were 2.14 and 2.43 for Disengagement and Exhaustion, respectively. Burnout thresholds were met by 54 and 66%of respondents for Disengagement and Exhaustion, respectively, with 86% meeting thresholds for either and 66% for both. The mean Disengagement scores were descriptively highest for nurses (2.89) and lowest for physicians (2.82), whereas mean Exhaustion scores were descriptively highest for psychologists (2.62) and lowest for other medical groups (2.54), however, there was no significant difference in the scores between different HCWs groups (see Table 2).

**Table 2:** Mean values of the level of Exhaustion and Disengagement and other variables in the groups of nurses, physicians, psychologists, and other health professions.

	**Group**										
**Professional**	**Physicians**		**Nurses**		**Psychologists**		**Other**				
**Groups**	***M***	***SD***	***M***	***SD***	***M***	***SD***	***M***	***SD***	***F***	***df***	***p***
Disengagement	2.82	0.50	2.89	0.53	2.88	0.50	2.86	0.41	0.40	3.313	0.75
Exhaustion	2.59	0.55	2.56	0.52	2.62	0.48	2.54	0.55	0.25	3.313	0.86
Positive emotions	3.76	1.05	4.20	1.28	3.82	0.98	3.88	1.32	3.07	3.306	0.03
Negative emotions	3.87	1.57	4.06	1.43	3.75	1.38	4.03	1.66	0.66	3.305	0.58
Depression symptoms	7.31	5.83	9.84	6.36	5.65	4.95	9.21	6.64	7.93	3.357	0.001
GAD symptoms	6.26	5.44	8.82	5.80	5.43	4.41	9.23	6.36	7.73	3.339	0.001

M, mean value; SD, standard deviation; F, analysis of variance value; df, degrees of freedom; p, statistical significance.

### Between-group comparison

**Figure 1:**
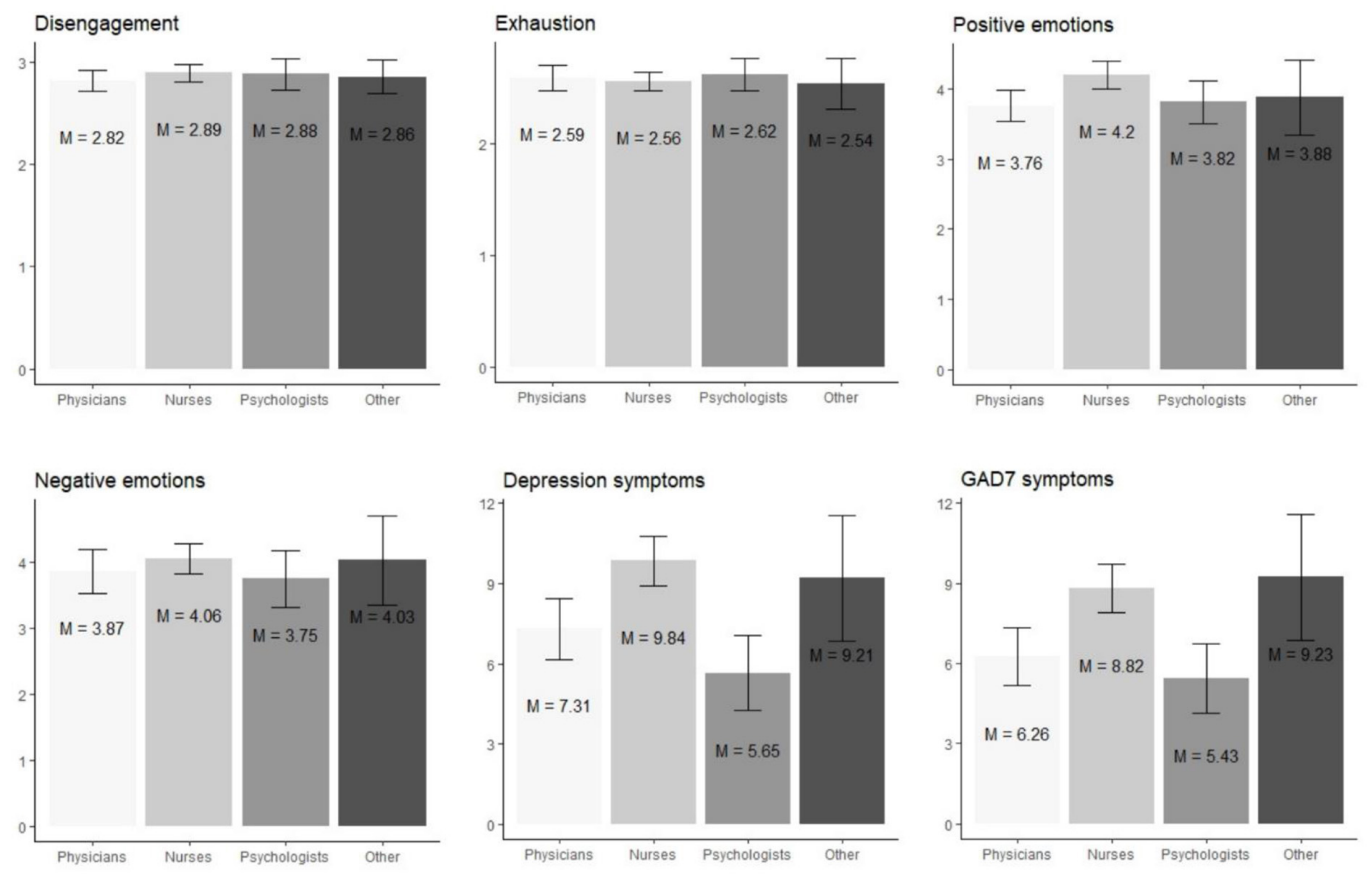
Visual comparison of levels of Exhaustion, Disengagement, and other variables in the groups of nurses, physicians, psychologists, and other health professions. Mean values are represented by bars and standard deviations are visualized using error bars.

Table 2 and Figure 1 presents mean values of the level of Exhaustion and Disengagement, and other measures in the HCWs professional groups with the values of one-way ANOVA.

No statistically significant between-group differences regarding Disengagement and Exhaustion were detected. However, there were differences in terms of depression symptoms, GAD symptoms, and positive emotions. The average level of generalized anxiety and depression in our sample reached 7.66 (*SD* = 5.78) and 8.55 (*SD* = 6.31), respectively. The threshold of moderate or severe symptoms of generalized anxiety [≥10 points; (24, 30)] was met by 34% of the participants of the current study, while the threshold for depression [>12 points, (31)] was met by 24% of the respondents. 19% of the participants fulfilled moderate or severe intensity of generalized anxiety and depression.

The level of generalized anxiety and depression varied among different groups of HCWs. The intensity of depression symptoms was significantly higher in the group of nurses than in the group of physicians, *p* < 0.01, and in the group of psychologists, *p* < 0.001. The intensity of GAD symptoms was also significantly higher in the group of nurses than in the group of physicians, *p* < 0.01, and in the group of psychologists, *p* < 0.001. The intensity of GAD symptoms was also significantly higher in the group of other health workers than in the group of physicians, *p* < 0.05, and in the group of psychologists, *p* < 0.05. The intensity of positive emotions was significantly lower in the group of physicians than in the group of nurses, *p* < 0.05. The rest of the differences were not statistically significant.

### Positive and negative emotions, depressive symptoms, GAD symptoms, and economic status as predictors of burnout

In the first step, partial Pearson correlation analysis of burnout, psychopathological symptoms, experienced emotions, economic status, and the rest of measured variables was performed (Table 3). Participants' age was used as a controlled variable. Gender was not included as a control variable due to the fact that the majority of participants were women (89%) and such a distribution did not allow for a reliable analysis of gender effects.

**Table 3:** Partial Pearson correlations of depressive symptoms, generalized anxiety symptoms, positive and negative emotions, and burnout controlling for age, redeployment to a different facility, COVID-19 dedicated workplace, work reorganizations, COVID-19 infection, psychological help at workplace, and economic status.

	**1**	**2**	**3**	**4**	**5**	**6**
1. Depressive symptoms	-	0.73^***^	−0.39^***^	0.61^***^	0.31^***^	0.51^***^
2. Generalized anxiety	0.73^***^	-	−30^***^	0.69^***^	0.22^***^	0.46^***^
3. Positive emotions	−0.39^***^	−0.30^***^	-	−0.39^***^	−0.44^***^	−0.45^***^
4. Negative emotions	0.61^***^	0.69^***^	−0.39^***^	-	0.36^***^	0.54^***^
5. Disengagement	0.31^***^	0.22^***^	−0.44^***^	0.36^***^	-	0.57^***^
6. Exhaustion	0.51^***^	0.46^***^	−0.45^***^	0.54^***^	0.57^***^	-

*** p < 0.001;

All controlled variables except for age and economic status (i.e. redeployment to a different facility, COVID-19 dedicated workplace, work reorganizations, COVID-19 infection, and psychological help at workplace) were dummy coded (0-no; 1-yes) dichotomous variables.

Next, in order to verify whether positive and negative emotions, depressive symptoms, GAD symptoms, and economic status are statistically significant predictors of burnout, linear regression analysis was performed. Tables 4, 5 present results acquired in the model for Disengagement. Due to a very strong correlation between the GAD and depressive symptoms, *r*(344) = 0.80, *p* < 0.001, they were analyzed in two separate regression models.

**Table 4:** Positive and negative emotions, depressive symptoms, and economic status as predictors of Disengagement.

**Predictors**	***B***	***95% CI***	***Beta***	***t***	***p***
Participants' age	< 0.001	−0.002, 0.006	−0.04	−0.83	0.41
Redeployment to a different facility	0.01	−0.20, 0.189	0.00	0.10	0.92
COVID-19 dedicated workplace	0.05	−0.18, 0.07	0.04	0.83	0.41
Work reorganizations	0.08	−0.19, 0.03	0.08	1.48	0.14
COVID-19 infection	0.02	−0.15, 0.11	0.02	0.31	0.75
Psychological help at workplace	−0.06	−0.04, 0.17	−0.06	−1.22	0.22
Depression	0.01	−0.02, 0.01	0.07	0.98	0.33
Positive emotions	−0.15	0.10, 0.20	−0.35	−6.16	0.001
Negative emotions	0.06	−0.11, −0.02	0.18	2.71	0.007
Economic status	−0.06	0.00, 0.11	−0.10	−1.96	0.05

B, unstandardized regression coefficient; 95% CI, 95% confidence intervals for B; Beta, standardized regression coefficient; t, test for statistical significance of predictor; p, statistical significance.

**Table 5:** Positive and negative emotions, GAD symptoms and economic status as predictors of Disengagement.

**Predictors**	***B***	***95% CI***	***Beta***	***t***	***p***
Participants' age	0.00	−0.002, 0.01	−0.06	−1.13	0.26
Redeployment to a different facility	0.01	−0.21, 0.18	0.01	0.14	0.89
COVID-19 dedicated workplace	0.05	−0.18, 0.08	0.04	0.78	0.44
Work reorganizations	0.09	−0.20, 0.02	0.08	1.61	0.11
COVID-19 infection	0.02	−0.15, 0.10	0.02	0.37	0.71
Psychological help at workplace	−0.08	−0.02, 0.19	−0.08	−1.57	0.12
GAD	−0.01	−0.01, 0.02	−0.07	−1.02	0.31
Positive emotions	−0.15	0.11, 0.20	−0.36	−6.56	0.001
Negative emotions	0.09	−0.14, −0.04	0.26	3.62	0.001
Economic status	−0.06	0.01, 0.12	−0.11	−2.25	0.03

B, unstandardized regression coefficient; 95% CI, 95% confidence intervals for B; Beta, standardized regression coefficient; t, test for statistical significance of predictor; p, statistical significance.

In the model with depressive symptoms as predictor, intensity of positive emotions and economic status were negatively related to Disengagement, whereas intensity of negative emotions was positively related to it. Intensity of positive emotions explained 12% of Disengagement variance, intensity of negative emotions explained 2% of Disengagement variance, and economic status explained 1% of variance. The model controlled for participants' age, redeployment to a different facility, COVID-19 dedicated workplace, work reorganizations, COVID-19 infection, psychological help at workplace, and depression level.

In the model with GAD as predictor, the model explained more of Disengagement variance: intensity of positive emotions explained 16%, intensity of negative emotions explained 3%, and economic status explained 1% of Disengagement variance, while controlling for the rest of variables.

Tables 6, 7 present results acquired in the model for Exhaustion.

**Table 6:** Positive and negative emotions, depression symptoms, and economic status as predictors of Exhaustion.

**Predictors**	***B***	***95% CI***	***Beta***	***t***	***p***
Participants' age	0.00	−0.002, 0.01	−0.05	−0.99	0.32
Redeployment to a different facility	−0.08	−0.11, 0.26	−0.04	−0.83	0.41
COVID-19 dedicated workplace	0.04	−0.16, 0.08	0.03	0.65	0.52
Work reorganizations	0.05	−0.15, 0.06	0.04	0.90	0.37
COVID-19 infection	−0.05	−0.07, 0.17	−0.04	−0.86	0.39
Psychological help at workplace	−0.01	−0.09, 0.11	−0.01	−0.18	0.86
Depression	0.02	−0.03, −0.01	0.24	3.92	0.001
Positive emotions	−0.11	0.07, 0.16	−0.25	−4.85	0.001
Negative emotions	0.11	−0.15, −0.07	0.30	5.10	0.001
Economic status	−0.01	−0.05, 0.06	−0.01	−0.29	0.77

B, unstandardized regression coefficient; 95% CI, 95% confidence intervals for B; Beta, standardized regression coefficient; t, test for statistical significance of predictor; p, statistical significance.

**Table 7:** Positive and negative emotions, GAD symptoms, and economic status as predictors of Exhaustion.

**Predictors**	***B***	***95% CI***	***Beta***	***t***	***p***
Participants' age	0.00	−0.002, 0.01	−0.05	−1.06	0.29
Redeployment to a different facility	−0.06	−0.13, 0.25	−0.03	−0.63	0.53
COVID-19 dedicated workplace	0.03	−0.16, 0.09	0.03	0.55	0.58
Work reorganizations	0.07	−0.17, 0.03	0.06	1.32	0.19
COVID-19 infection	−0.05	−0.07, 0.17	−0.04	−0.85	0.40
Psychological help at workplace	−0.02	−0.08, 0.12	−0.02	−0.39	0.70
GAD	0.01	−0.03, −0.002	0.16	2.36	0.02
Positive emotions	−0.13	0.08, 0.17	−0.28	−5.59	0.001
Negative emotions	0.12	−0.17, −0.07	0.33	4.94	0.001
Economic status	−0.01	−0.04, 0.01	−0.02	−0.46	0.65

B, unstandardized regression coefficient; 95% CI, 95% confidence intervals for B; Beta, standardized regression coefficient; t, test for statistical significance of predictor; p, statistical significance.

Intensity of negative emotions, depressive symptoms, and GAD symptoms were positively related to Exhaustion, whereas intensity of positive emotions was negatively related to Exhaustion. In the model including depression symptoms, these symptoms explained 28%, positive emotions explained 7%, and negative emotions explained 5% of Exhaustion variance, while controlling for the same variables as earlier (participants' age, redeployment to a different facility, COVID-19 dedicated workplace, work reorganizations, COVID-19 infection, psychological help at workplace). In the model including intensity of GAD symptoms, these symptoms explained 22% of Exhaustion variance, positive emotions explained 10% of Exhaustion variance, and negative emotions explained 5%, whereas economic status was not related to the level of Exhaustion, while controlling for the rest of variables.

## Discussion

The aim of the current study was to evaluate the level of burnout among HCWs during COVID-19 pandemic and to study predictors of burnout. We found that burnout thresholds for both Disengagement and Exhaustion were met by 66% of HCWs. This is comparable to the rates (67% HCWs) reported by Denning et al. (8) in the multinational cross-sectional study assessing 3,537 healthcare workers, including 232 HCWs from Poland in the similar period of COVID-19 pandemic with the same tool for measuring burnout. Our mean scores of burnout dimensions, 2.14 and 2.43 for Disengagement and Exhaustion, respectively, are also comparable to those reported in Singapore in the same period of COVID-19 pandemic with mean Exhaustion and Disengagement scores of 2.38 and 2.25, respectively (32). These rates suggest that the COVID-19 pandemic and its burdens were related to elevated rates of burnout amongst HCWs in Poland. When we used the Polish burnout norms for social service workers established in 2016 (20), 76% of our HCWs presented a moderate (cutoff 1.91) or high (cutoff 2.75) level of Exhaustion and 54% experienced moderate (cutoff 1.89) or high (cutoff 2.72) level of Disengagement. Comparison of the burnout scores obtained in our study and the mentioned study from 2016 which was conducted before pandemic in professions of social services including HCWs (20), suggests presumably higher levels of Exhaustion but lower mean of Disengagement in our sample. Note, that current research did not include repeated measurements and the pre-pandemic scores were obtained in professions of social services in the other study, in different time and context, which make any direct comparisons questionable. Still, it may indicate the direction of relationships and suggest that persistent, increased tension and demands that emerged with medical resources and services placed at their maximum capacity during COVID-19 pandemic may affect burnout level and Exhaustion dimension in particular [see also (33)]. At the same time, these demands placed on HCWs required unprecedented engagement in their work, which if accompanied by adaptive self-regulation of emotion might buffer the elevation of Disengagement component of burnout (34) at this stage of COVID-19 pandemic in Poland, the hypothesis that requires verification in future studies. Still, 54% of HCWs in the present study met the burnout thresholds for Disengagement and 66% for Exhaustion.

Regarding predictors of burnout, we hypothesized that intensity of negative emotions along with depressive and anxiety symptoms will positively predict both dimensions of burnout, whereas positive emotions and higher economic status will be negatively related to them. Our results confirm some of our predictions. Indeed, when controlling for participants' age, and several variables related to work organizations during the pandemic (e.g., redeployment to a different facility, work reorganizations, COVID-19 infection), intensity of negative emotions was positively related to both Disengagement and Exhaustion. However, depression and GAD symptoms were positively related to Exhaustion only. Intensity of positive emotions was a negative predictor of both dimensions of burnout, and economic status was a negative predictor of Disengagement, but not Exhaustion.

A number of previous studies showed positive relationships between depression, anxiety, and burnout [e.g. (8, 32)]. Also a recent meta-analysis supported this observation and indicated that there is no conclusive overlap between burnout and depression and burnout and anxiety (22). The finding that Exhaustion is related to depression and anxiety specifically was also reported by other researchers (35, 36). We did not find, however, any study that took into account broader constructs of negative emotions or positive emotions of HCWs as predictors of burnout. Therefore, the novel aspect of the present study, among others, is to evidence the importance of experienced emotions in predicting burnout.

Experiencing negative emotions (e.g., anger, sadness) contributed in our research mainly to the Exhaustion dimension of burnout. This is understandable as Exhaustion is a result of persistent, chronic tension caused by physical, emotional, and cognitive demands of a job that is accompanied by negative emotions (21). Negative emotions explained a substantial part of Exhaustion variance (33%), much more than other predictors: depression and positive emotions (4 and 7% respectively). These results show that the use of a broader spectrum of negative emotions (as in our research model) than just anxiety and depression, enables a better understanding of burnout. Earlier studies showed that negative emotions experienced by people under stress narrow down their thought action repertoire (37). Negative emotions experienced by HCW's might contribute to maladaptive strategies to cope with increased job demands, such as inflexible coping and self-undermining, which may in consequence impair self-regulation abilities [see also (34)].

Positive emotions were in our research the strongest predictor of Disengagement—they explained 22% of its variance. It suggests that experiencing positive emotions may reduce withdrawal toward HCWs clients and co-workers and possibly support HCWs well-being. The role of positive emotions shown in our research, broadly speaking, is consistent with the assumptions of the Broaden and Build Model of Positive Emotions proposed by Fredrickson (38, 39). According to this model, positive emotions enable adaptive and constructive functioning. Positive emotions broaden perception in a way that helps to have a wider and appreciative perspective, and therefore build additional resources to cope with stressful situations. The search for new resources to apply seems to be incongruent with Disengagement, and can be viewed as protective in the light of the recently reformulated, by inclusion of self-regulation, Job Demands–Resources theory (34). Factors enabling the rebuilding of resources are particularly important in situations of chronic and uncontrolled stress, which was the case during the period of the COVID-19 pandemic when our study was conducted.

In our study, generalized anxiety and depression were noted in 34 and 24% of participants, respectively, which is similar to depression rates in other studies, but at the same time higher with respect to anxiety (15, 19, 40). We found that anxiety was more prevalent than depression [see also (8)]. Respondents with depression or generalized anxiety were likely to also have symptoms of burnout, which is consistent with a number of studies, including a recent meta-analysis (40). The level of generalized anxiety and depression varied among different groups of HCWs, with nurses having significantly higher rates of psychopathological symptoms than physicians and psychologists, as well as other healthcare workers having significantly higher levels of GAD symptoms than physicians and psychologists. Nurses and other healthcare workers also had higher means of depression and generalized anxiety in comparison to the means of the Polish population measured in May, June, July, and December 2020 (41, 42).

Another novel aspect of the present study was the inclusion of economic status as a possible predictor of burnout. We found that economic status was a negative predictor of Disengagement and explained over two times more variance of this dimension than negative emotions. It implies that good economic status was associated with less withdrawal among HCWs. Other studies showed that organizational interventions such as alterations to workload or changes to working practices produce longer-lasting effects than individual approaches to burnout prevention (43). The current findings suggest, in addition, that good financial situation of HCWs may have beneficial effects on burnout prevention, particularly its Disengagement facets. Future studies need to evaluate whether this burnout predictor is valid also for the HCWs working in other countries, as low level of healthcare financing from public funds and low incomes of HCWS is a specific ongoing problem in the Polish healthcare system (44).

Correlational analysis in our research revealed a few weak associations of some variables with burnout. These relationships were too weak to include those measures in our regression analysis for establishing burnout predictors, but are still worth commenting on. First, there was a correlation of test-confirmed COVID-19 infection in HCWs and elevated Exhaustion, which could be expected as burnout was reported to be associated with fear of exposure to or transmission of SARS-CoV-2 virus (12). Quite unexpectedly, we were not able to confirm an association between burnout and staff redeployment, which is at odds with some previous studies (8). There was, however, a relationship between burnout dimensions and reorganizations at work in the expected direction showing that reorganizations at work were related to the increase in Exhaustion and Disengagement.

### Limitations and future directions

The current study has some limitations concerning the cross-sectional design, which precludes causality inference. In addition, the sample was acquired through posts and advertisements and is limited to Polish HCWs, therefore, generalizability of the results is limited. Another limitation is that the current study relied upon self-report scales, which are prone to a number of inherent confounds, such as biases in recall, social desirability or the participants' mood (45). Future studies may benefit from diary and ecological momentary assessment methods that enable researchers assessing the ongoing experience of examined individuals in their natural environment while reducing biases in recall (46). Finally, our sample was not representative. Still, we believe, the current study contributes to the literature on the subject of burnout in health professionals in a number of ways, including evidencing the importance of experienced emotions and economic status as possible predictors of burnout.

## Conclusions and implications

Previous research showed that burnout is related to increased risks of both physical and psychological long-term detrimental effects in health professionals, along with their increase in sick leave, absenteeism, job withdrawal, and poor work efficiency (44). Therefore, elevated rates of burnouts in HWCs during prolonged stressful situations such as the COVID-19 pandemic might reduce the capacity of health systems to effectively cope with the increased demand of care (47, 48). As timely recognition of burnout problems is crucial for implementation of effective prevention or therapeutic programs, it is important to promote monitoring of the HCWs mental health status and to deliver prevention programs including psychological first aid for individuals at risk. Results of the current study suggest that presence of depression and anxiety along with negative emotions should be taken into account in interventions aimed at preventing the development of burnout in HCWs. Our findings suggest also that such interventions should be aimed not only at decreasing depression and negative emotions, but also at increasing positive emotions. It is worth noting that positive and negative affect are independent (49), thus reduction of negative emotions does not mean that positive emotions increase.

The important role of experienced emotions as a predictor of burnout in a situation of chronic stress prompts us to refer to James Gross's Emotional Regulation model (50). According to this model, there is a wide range of strategies that may help to modify experienced emotions, and considering this model may be helpful in burnout prevention (43, 48, 51). Such burnout interventions and prevention programs should take into account a broad range of factors that possibly cause burnout including those delineated in the present study. Such interventions should also implement a variety of strategies both on an organizational as well as on an individual level [see (51) for a meta-analysis]. The findings of the current study indicate also that a good economic status of HCWs may play an important role in the prevention of burnout and should be taken into consideration by health providers.

## Data availability statement

The datasets for this study can be found in the [OSF] [https://osf.io/3w5ak/].

## Ethics statement

The studies involving human participants were reviewed and approved by the Institutional Review Board of the Faculty of Psychology, University of Warsaw. The patients/participants provided their written informed consent to participate in this study.

## Author contributions

PH, MG, KH, GK, EP, KB-M, and EŁ contributed to conception and design of the study. PH and NW organized the database and performed the statistical analysis. PH wrote the first draft of the manuscript. NW, MG, KH, GK, EP, KB-M, and EŁ were involved in writing sections of the manuscript. All authors contributed to manuscript revision, read, and approved the submitted version.
